# MFRED, 10 second interval real and reactive power for groups of 390 US apartments of varying size and vintage

**DOI:** 10.1038/s41597-020-00721-w

**Published:** 2020-11-09

**Authors:** Christoph J. Meinrenken, Noah Rauschkolb, Sanjmeet Abrol, Tuhin Chakrabarty, Victor C. Decalf, Christopher Hidey, Kathleen McKeown, Ali Mehmani, Vijay Modi, Patricia J. Culligan

**Affiliations:** 1 Data Science Institute, Columbia University, New York, USA; 2 Earth Institute, Columbia University, New York, USA; 3 Department of Mechanical Engineering, Columbia University, New York, USA; 4 Department of Computer Science, Columbia University, New York, USA; 5 Department of Civil Engineering and Eng. Mechanics, Columbia University, New York, USA

**Keywords:** Energy supply and demand, Energy modelling

## Abstract

Building electricity is a major component of global energy use and its environmental impacts. Detailed data on residential electricity use have many interrelated research applications, from energy conservation to non-intrusive load monitoring, energy storage, integration of renewables, and electric vs. fossil-based heating. The dataset presented here, Multifamily Residential Electricity Dataset (MFRED), contains the electricity use of 390 apartments, ranging from studios to four-bedroom units. All apartments are located in the Northeastern United States (IECC-climate-zone 4 A), but differ in their heating/cooling system and construction year (early to late 20^th^ century). To adhere to privacy guidelines, data were averaged across 15 apartments each, based on annual electricity use. MFRED includes real and reactive power, at 10-second resolution, for January to December 2019 (246 million data points). The annual average real power per apartment is 343 W (3.27 W/m^2^ of floor area), with strong variation between seasons and apartment size. Considering its large number of apartments, high time resolution, real and reactive power, and 12-month duration, MFRED is currently unique for the multifamily-sector.

## Abstract (summary)

**Table:** 

Measurement(s)	Electricity • real power measurement at 10-second interval • reactive power measurement at 10-second interval
Technology Type(s)	electricity meter • micrometer system
Factor Type(s)	heating/cooling system in apartment • construction year of apartment • apartment’s floor area • apartment’s number of rooms
Sample Characteristic - Environment	apartments in multi-family residential building • residential building
Sample Characteristic - Location	contiguous United States of America

Machine-accessible metadata file describing the reported data: 10.6084/m9.figshare.13102490

## Background & Summary

Electricity consumption in buildings is a major component of global energy use and its environmental impacts, including greenhouse gas (GHG) emissions. In the United States, the residential building sector in 2017 consumed 32% of all electricity^1^, an amount responsible for 9% of all domestic GHG emissions^2^. Globally, electricity consumption is expected to grow, however with increasing portions generated by renewables sources such as solar and wind^3^.

Understanding residents’ electricity use has many interrelated research applications, including: (i) determining the potential for energy conservation through resident feedback^4–8^; (ii) non-intrusive monitoring (NILM) of appliance-level loads via disaggregation^9–11^; (iii) predicting building electricity use^12–14^, including with agent-based models^15–17^; (iv) leveraging a building’s thermal characteristics to optimize its electricity profile used for heating and cooling^18,19^; (v) switching heating systems from fossil fuels to electricity, e.g., via heat pumps^20^; (vi) assessing the techno-economic feasibility for distributed electric storage to facilitate integration of intermittent renewables on the grid^21–23^, including electric vehicles^24,25^; (vii) quantifying phantom loads, e.g., from electronics in standby mode^11^; (viii) detecting occupancy patterns^26^; and (ix) novel market mechanisms and associated technology solutions to enhance the integration from buildings into smart grids^27^, e.g., for applications in Transactive Energy Networks^28^.

Here, we present the Multifamily Residential Electricity Dataset, henceforth MFRED (Data Citation 1). MFRED features 10-second resolution real and reactive power in 390 apartments, ranging in size from studios to four bedrooms. To place MFRED into context, we note that for US single family homes (detached or attached dwellings) a number of publicly available datasets on electricity use already exist. These typically feature either a large sample size but at low time resolution (e.g., the RBSA study with 100 homes at the standard 15 minute resolution^29^) or a small sample size at high time resolution (e.g., the REDD study with 10 homes at 1-second or higher time resolution^10^). Outside the US, examples include REFIT in the United Kingdom (electricity data of 20 houses, including appliance level, at 8-second time resolution over 2 years)^30^, AMPds in Canada (1-minute interval electricity loads of a Canadian house, including 21 separate submeter circuits, over 2 years)^31^, and SustData in Portugal (sub-second interval electricity loads of 6 single family homes and 17 apartments over ~9 months)^32^. In contrast, for apartments in US multifamily buildings, which are increasingly common, very few datasets have been made public. One example is data of a student housing-style condominium building in Los Angeles, California, with hourly resolution of 118 apartments over 8 months^33^. With these considerations in mind – diverse multi-family units, sample size, 10-second time resolution, both real and reactive power, and a full year of data – MFRED appears unique.

## Methods

### Data collection

With authorization from Columbia University’s *Institutional Review Board*, the electric consumption data in MFRED were collected by a standard installation of a Siemens® *embedded micro metering system*^34^ with *SEM3* controllers and *9410* data loggers, running in combination with *WinPM* data collection software^35^ (henceforth Real-Time Metering, RTM). The hardware and software installations were carried out by licensed contractors.

The RTM for each apartment (sense, log, communicate) was installed in the buildings' utility rooms, in proximity to the existing utility meters and apartment circuit breakers in each building (rather than inside each apartment), with fifty-amp split-core current transformers on each pole going to an apartment, to measure real-time currents. Currents are relayed to a controller (firmware version 2.3.7.AE; 1–2 controller(s) per building), which computes real and reactive powers using the voltage probed at the building’s main circuit breaker. Some apartments are wired with only one pole and others with two poles at different phase (“3 Phase Wye” configuration). Each controller transmits the power and energy data (see section *Data record glossary*) to a data logger (firmware version 001.003.001), which timestamps the data and reports them to a central server via the building’s Ethernet. The data loggers store about 18 hours of data in onboard memory, acting as a buffer in case of temporary Ethernet outages. The server runs the data collection software (*version 7.1*) on a *Microsoft® Windows Server 2012R2* virtual machine. The data collection software stores the data in a *Microsoft SQL* database on the server.

Various test routines were carried out to ensure that the data for each apartment were properly communicated to the *SQL* database. For example, during installation of the RTM, this included measuring the amperage of every pole with a separate, handheld ampere meter and cross-validating this amperage against the respective data in the controllers. Additional test routines, including for meter accuracy, are described in *Technical Validation*.

### Data de-identification and 15/15 rule

All data in MFRED have been fully de-identified and thus carry no identifiable information such as names of residents, building addresses, or apartment numbers. To further reduce the risk of possible re-identification or privacy breaches, and in consultation with the project management at the US Department of Energy and data privacy and re-identification experts (see *Acknowledgements*), MFRED follows the data aggregation standard recommended for publishing utility data in the State of New York, commonly referred to as the 15/15 rule^36^. Briefly, this standard recommends that electricity data not be made public at the level of individual apartments but only as aggregates of 15 (or more) apartments each, with the additional requirement that no individual apartment comprise more than 15% of the respective aggregate (see section *Data record/Apartment groups* for details).

## Data Records

### Building types and heating/cooling systems

The 390 apartments in MFRED (dataset available on *Harvard Dataverse*^37^) are located across over a dozen buildings in the borough of Manhattan, New York, NY, USA (IECC climate zone 4A^38^). All 390 apartments are rented, and the vast majority of residents pay for their electricity through their own contract with the local utility (rather than through their rent).

The buildings were chosen to represent typical Manhattan residential building stock: 79% of the buildings in MFRED were constructed prior to 1940, 7% between 1940 and 1980, and 14% post 1980. The average size of apartments in MFRED (which ranged from studios to four-bedroom units) is 105 m^2^ (standard deviation: 48 m^2^). For comparison, the entire Manhattan residential and mixed-residential building stock is 86% pre-1940, 6% 1940–1980, and 8% post-1980, with an average apartment size of 92 m^2^ (standard deviation: 64 m^2^)^39^.

Of the apartments in MFRED, 89% have heating supplied centrally (steam or hot water), but the air conditioning is provided by the residents’ own appliances (typically installed in the windows). This means that, with the exception of supplementary personal space heaters or heating blankets, heating in these apartments does not contribute to the electricity data reflected in MFRED, whereas air conditioning does. The remaining 11% of apartments use various types of packaged terminal air conditioning units (PTAC), with the majority such that the bulk of the heating and cooling load is supplied centrally, thus not contributing to an apartment’s own electric loads. Consequently, the majority of apartments in MFRED exhibit higher electric power draw during summer months, as a function of weather conditions. In contrast, the power draw during winter months depends only marginally on weather conditions.

### Apartment groups

Following the 15/15 rule^36^ (above), we organized the 390 apartments into 26 groups of 15 apartments each (Table 1). These groups are identified by the prefix “AG01” through “AG26” in the column headers for each data record in MFRED. To simplify the use of MFRED, the data for each apartment group show the average (not the sum) of each electricity metric at each time step (Table 2), averaged across the 15 apartments that belong to each AG.

**Table 1:** Overview of the 26 apartments groups (“AG”) in MFRED.

Apt. group	Time-averaged real power (*σ*) [W]	Number of bedrooms (*σ*)	Number of all rooms (*σ*)	Apt. area (*σ*) [m^2^]
AG01	42 (19)	1.2 (0.7)	3.2 (1.3)	62 (28)
AG02	81 (7)	1.3 (0.5)	3.9 (1.8)	77 (36)
AG03	105 (5)	1.3 (0.6)	3.3 (1.3)	65 (29)
AG04	119 (4)	1.1 (0.3)	3.1 (1.0)	58 (18)
AG05	141 (6)	1.5 (0.6)	4.2 (1.5)	91 (41)
AG06	156 (4)	1.3 (0.5)	3.8 (1.4)	77 (33)
AG07	166 (4)	1.4 (0.6)	4.2 (1.5)	85 (42)
AG08	179 (4)	1.3 (0.6)	3.8 (1.5)	77 (38)
AG09	189 (3)	1.4 (0.6)	3.7 (1.7)	86 (43)
AG10	203 (4)	1.3 (0.6)	3.7 (1.4)	81 (29)
AG11	216 (4)	1.9 (0.8)	4.9 (1.8)	102 (43)
AG12	228 (5)	1.4 (0.5)	4.0 (1.3)	81 (27)
AG13	245 (5)	1.7 (0.7)	4.2 (1.4)	83 (35)
AG14	267 (7)	1.6 (0.6)	4.1 (1.5)	73 (30)
AG15	289 (8)	1.9 (0.6)	5.1 (1.4)	111 (29)
AG16	317 (9)	2.1 (0.7)	5.8 (1.1)	119 (36)
AG17	340 (7)	2.0 (0.8)	5.7 (1.4)	115 (36)
AG18	366 (8)	1.9 (0.6)	5.1 (1.4)	114 (43)
AG19	393 (8)	2.1 (0.5)	5.3 (1.4)	118 (46)
AG20	434 (12)	2.3 (0.6)	5.9 (1.1)	137 (32)
AG21	470 (17)	2.8 (0.6)	6.4 (1.2)	159 (39)
AG22	528 (20)	2.5 (0.8)	6.0 (1.1)	144 (42)
AG23	589 (29)	2.0 (0.7)	5.4 (0.9)	116 (30)
AG24	714 (36)	2.9 (0.7)	5.8 (1.1)	160 (45)
AG25	874 (42)	2.6 (0.8)	6.5 (1.0)	165 (27)
AG26	1255 (425)	2.7 (0.7)	6.5 (0.9)	164 (41)
**Average**	**343**	**1.8**	**4.8**	**105**

AGs are sorted from lowest to highest annual electricity consumption. Real power, number of rooms, and floor area are specified per average apartment (“all rooms” specifies bedrooms, living/dining rooms, kitchens, and bathrooms). Standard deviations (σ) of the respective data are shown as well, in order to provide MFRED users with the variability of apartments in each AG.

**Table 2:** Data record glossary for MFRED.

Column header	Explanation, unit, accuracy (where applicable)
DateTimeUTC	Time stamp of each data entry, at UTC (coordinated universal time, also known as Greenwich Mean Time), i.e. not adjusted for daylight saving; csv files show time in format: yyyy:mm:dd HH:mm:ss (24 h format)
kW*	Instantaneous real power [kW] per apartment, observed at DateTimeUTC; accuracy is ± 1%, however the minimum detection threshold is 0.003 kW
kVAR*	Instantaneous reactive power [kW] per apartment, observed at DateTimeUTC; accuracy is ±1%; inductive loads draw positive, whereas capacitive loads draw negative reactive power
kWh*	Cumulative electricity consumed [kWh] per apartment, between 01-Jan-2019 5:00:00 UTC (i.e., first time stamp in MFRED) and DateTimeUTC
kVA	Instantaneous apparent power is not included in MFRED but can be calculated via the power triangle^40^ as follows: kVA = sqrt(kW² + kVAR²)
Phase angle	Instantaneous phase angle (PA) is not included in MFRED but can be calculated via the power triangle^40^ as follows: tan(PA) = kVAR/KW

*For each of the 26 apartment groups (AG), electricity metrics are averaged across the 15 apartments in each group. The group that each column refers to is indicated by the prefix “AG01”, “AG02”, …, “AG26” in the column headers. For ease of use, a grand average across all 390 apartments is also provided (indicated by the prefix “AGs01To26” in the column headers). Each metric therefore represents the kW, kVAR, or kWh of an average apartment (not the sum across apartments). The total real [reactive] power on the grid can be calculated by multiplying the given average kW [kVAR] value with the respective number of apartments.

In order to obtain AGs with similar electricity use in each group, we first sorted the 390 apartments by their total electricity consumption in 2019. AG01 comprises the 15 apartments with the lowest 2019 consumption, AG02 the next 15 apartments, etc. This approach also ensured that the 15% requirement of the 15/15 rule was not violated: For 23 of the 26 AGs, the highest single-apartment portion is 7%, and for the remaining 3 AGs it is 13%.

### Data record glossary

Table 2 lists each data record in MFRED along with its technical explanation. The acronyms kW, kVAR, and kWh follow standard terminology^40^. The kWh data record can be used to interpolate electricity use during the rare times that some apartment meters were offline (see section *Technical validation/Data gaps*). For an illustration of how to interpret the various metrics, see section *Seasonality and end-use types*.

### MFRED file organization

MFRED is organized into five csv files (Table 3). Four of these contain the data at 10-second time resolution, one file for each quarter of 2019. The number of rows per csv file was kept below 1 million, to allow for immediate analysis in standard spreadsheet software. The 5^th^ file shows the data of all 4 quarters in a single file, but at 15-min time resolution. Note that, for either time resolution, the real and reactive powers show *instantaneous* readings at each time step, not readings time-averaged for the period in-between time steps. As such, the 15-min file is simply an excerpt of the other 4 files, provided solely for the convenience of a single, smaller file for the entire year.

**Table 3:** Names and descriptions of MFRED files (Data Citation 1).

Period	Resolution	Name of csv file	File size (compressed)	Number of rows in csv file**	Avg. fraction of apts. reporting per time step
2019Q1	15-min	*n/a*	*n/a*	8,636	100%*
	10-sec	MFRED_Agg_10s_2019Q1.csv	514MB (171MB)	777,240	99.9998%
2019Q2	15-min	*n/a*	*n/a*	8,736	100%*
	10-sec	MFRED_Agg_10s_2019Q2.csv	530MB (173MB)	786,240	99.9999%
2019Q3	15-min	*n/a*	*n/a*	8,832	99.9512%
	10-sec	MFRED_Agg_10s_2019Q3.csv	537MB (180MB)	794,880	99.9515%
2019Q4	15-min	*n/a*	*n/a*	8,836	100%*
	10-sec	MFRED_Agg_10s_2019Q4.csv	550MB (177MB)	795,240	99.9995%
2019Q1-Q4	15-min	MFRED_Agg_15m_Q2019Q1-4.csv	24MB (9MB)	35,040	99.9878%
	10-sec	*n/a*	*n/a*	3,153,600	99.9877%

The “reporting per time step” metric is explained in section Technical validation/Data gaps.

* Indicates files with complete coverage (all 390 apartment meters reporting at every 15-min time step).

** All files contain one row for every 15 min [10 sec] time step during the covered time period.

### Benchmarking of annual average consumption

**Fig. 1:**
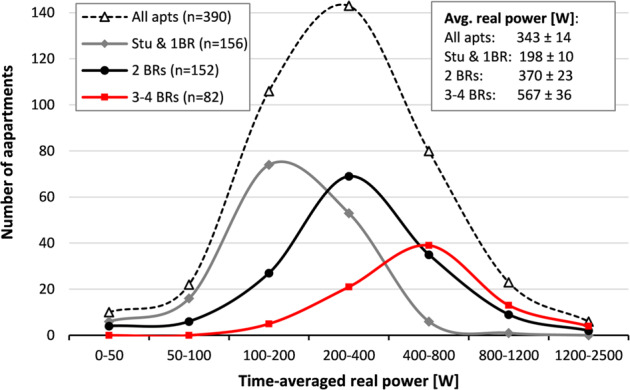
Histogram of 12-month time-averaged real power in the 390 apartments (Jan. through Dec. 2019), by apartment size (“BR” denotes number of bedrooms). Values in inset box show average ± SEM loads for each size class. Lines drawn between markers for each histogram bin are for visual purposes only.

Between 01 January 2019 and 31 December 2019, the time-averaged electricity use (real power) per apartment was 343 ± 14 W, or 8.2 ± 0.3 kWh per day (where 14 W [0.3 kWh] is the standard error of the mean across the 390 apartments, SEM). The time-averaged use varies strongly between apartments, from less than 50 W to ~2,500 W. As seen in Fig. 1, the variation is partly explained by apartment size. However, even among similarly-sized apartments, differences of a factor 3 or more in time-averaged use are common. Note that, based on electricity use patterns, it appears that some of the 390 apartments were unoccupied for part of the year. Since this is common for rental units in multi-family buildings (e.g., in-between previous and new tenants), the data of these apartments were included in the apartment group averages in MFRED.

An average real power of 343 ± 14 W per apartment is consistent with summary statistics from the *Residential Energy Consumption Survey* (RECS) data (latest available data are from 2015)^41^. According to that survey, apartment units in multifamily buildings of 5 or more units in the Northeastern United States had an average electricity use of 340 W (when excluding any electricity used for space or water heating)^42^.

Considering another benchmark, the average electricity use intensity (i.e., use per floor area) in the apartments in MFRED is close to that observed for single family homes, once adjusted for types of electricity use. In the United States *Residential Building Stock Assessment* (RBSA)^29^, one of the largest databases for single family homes in the United States, the annual average electricity is 1,497 W per home, corresponding to an average electricity use of 8.03 W/m^2^. However, 62% of this use is attributed to electricity used for heating, leaving 3.05 W/m^2^ for all other electricity use. The value of 3.05 W/m^2^ is close to the average electricity use intensity in MFRED, which is 3.27 W/m^2^ (Table 1). The 7% higher electricity use intensity in the apartments in MFRED is likely attributable to the following effects: (i) summer cooling loads in climate zone 4 A are higher than in the more temperate climates in the North Western United States that are reflected in RBSA; (ii) apartments, even if small, will use basic appliances such as a refrigerator, thus increasing the average electricity use per m^2^ of apartments vs. that of single family homes. In addition to these differences, apartments vs. single family homes may differ in lighting use per m^2^ or number of residents per m^2^, both of which would affect electricity use intensity.

### Seasonality and end-use types

**Fig. 2:**
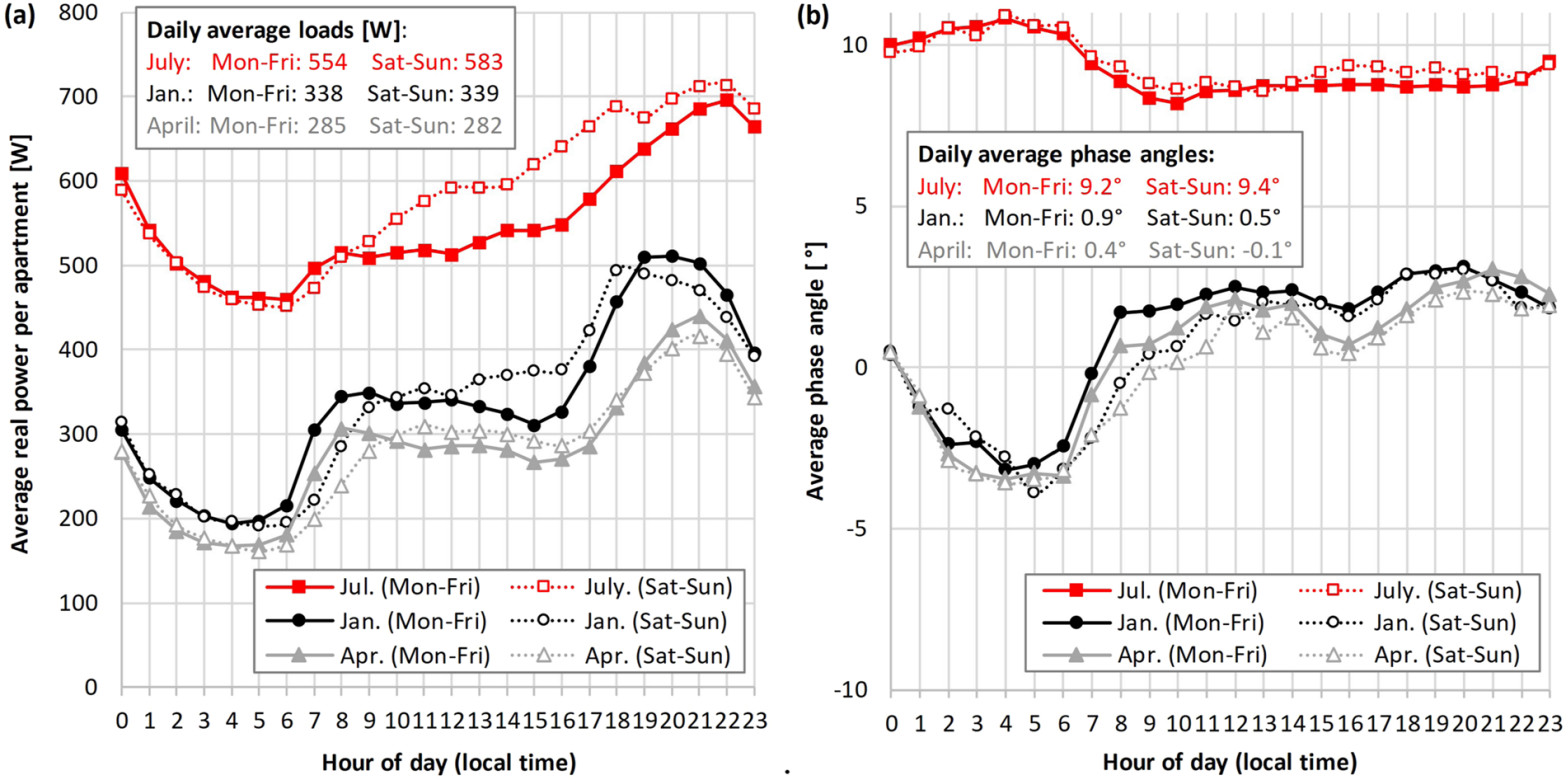
**(a)** Diurnal load profiles by season and weekday vs. weekend (2019). Each line shows the instantaneous real power at the full hour, averaged across all weekday/weekend days in the time period and across all 390 apartments. To ensure consistent representation of weekday vs. weekend, each time period starts on a Monday and covers exactly 28 days: 07 Jan.–03 Feb. for winter, 01 Apr.–28 Apr. for the shoulder season, and 08 Jul.–03 Aug. for summer. **(b)** Same as (**a**), but showing the phase angle, calculated from real and reactive powers as per Table 2.

Figure 2(a) shows the electricity use for different times of the year, with each time of year illustrated using a period of 28 days. These periods are in July 2019 (for summer), January 2019 (for winter), and April 2018 (for the shoulder season). The average real power is highest in July (554 W on weekdays, 583 W on weekends) because the majority of the MFRED apartments use electric window air conditioners rather than centrally supplied cooling. The average real power is lowest in April (285 W on weekdays, 282 W on weekends) when there is little need for either air conditioning or supplementary electric heaters. January has a slightly higher average real power than April (338 W on weekdays, 339 W on weekends), likely because residents use electric lighting for more hours of the day and some use supplementary electric heating. Still, for the majority of MFRED apartments heating is supplied centrally, so it does not have a significant impact on apartment-level electricity use.

Seasonal differences exist in the diurnal profiles as well. Most notably, the typical load increase in the early evening hours (17–20 h) is steeper in January than in April or July. In July, the diurnal profile, while showing higher use overall, also shows less variation of use during the day. This is expected in climate zone 4 A for the month of July, which features hot and humid weather conditions, prompting substantial use of air conditioning throughout the day and night.

A unique feature of MFRED is that it includes both real and reactive power. This allows MFRED users to separately identify three different types of loads: (i) Inductive loads (such as the electric motors in air conditioners), which draw positive reactive power and thus increase the phase angle of the total apartment load; (ii) capacitive loads (including some consumer electronics), which draw negative reactive power and thus decrease the phase angle; and (iii) resistive loads (such as incandescent light bulbs or space heaters), which draw no reactive power and thus reduce the absolute value of the phase angle (i.e., closer to zero)^40^. As an example of such an analysis, the phase angle shown in Fig. 2(b) provides insight into which types of appliances are predominantly used at different times of day and how this varies by season: The elevated phase angle in July is consistent with the above interpretation that the additional electricity use in July seen in Fig. 2(a) stems from increased use of air conditioners, which have large inductive loads. The negative phase angle observed at night in January and April indicates that electronics such as WiFi routers, internet modems, computers, and entertainment devices dominate the average apartment’s electricity use at these times. When such electronics are not actively being used (such as a television in stand-by mode), they are often referred to as phantom loads^11^.

## Technical Validation

### Apartment wiring, data consistency, and dynamic range

As laid out in more detail in *Methods*, all electricity data in MFRED were collected by a standard installation of a vendor-provided RTM. To validate that the metering system is running as expected, we carried out several test routines. One test routine, already described in *Methods*, ensured that each of the ~750 current transformers was wired to the correct RTM micro meter module. We carried out the following further test routines, with the focus on ensuring that each of the data fields in the *SQL* database imports the correct data from the respective controller.

#### Internal consistency of data

While only the kW, kVAR, and kWh data records are included in MFRED, the controllers record additional metrics for every apartment, including amperage, voltage, apparent power, and phase angle. Each controller displays these metrics on a separately accessible interface. The metrics are interrelated in what is commonly referred to as the power triangle^40^. For example, the phase angle can be inferred mathematically from kVAR and kW (Table 2). We used these mathematical relationships to cross-validate the metrics displayed by the controllers directly (e.g., phase angle) with the respective values calculated from the *SQL* database. This gave us very high confidence that all data fields in the *SQL* database import the correct data from each controller.

#### Dynamic range of metrics

We validated that all metrics fall into their expected ranges, specifically: (i) instantaneous real power is positive, ranging from 0 kW to about 15 kW per apartment (for large apartments, at some times of day); (ii) reactive power is negative or positive, but typically substantially smaller in absolute value than real power (as seen from the phase angles in Fig. 2(b)); (iii) average real power per floor area is consistent with benchmarks (see section *Data records/Benchmarking)*; and (iv) voltage per pole is ~120 V.

### Accuracy

**Fig. 3:**
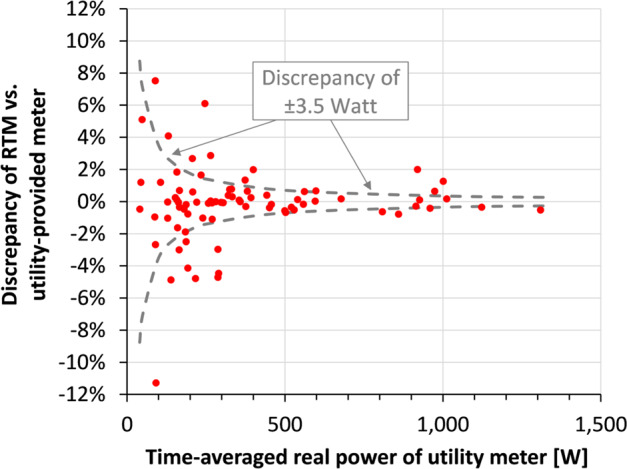
Results of meter accuracy test. The load-weighted average discrepancy in the 78 tested apartments is −0.05%, showing that there is no material systematic error of the RTM vs. the utility-provided meters. When considering only the absolute value of the discrepancy (i.e., irrespective of the sign), the load-weighted average is 0.92% (or 3.5 Watt), consistent with the manufacturer’s ± 1% accuracy rating for the RTM.

The manufacturer-rated relative accuracy of the RTM with split-core current transformers is ±1%^34^, with lower relative accuracy expected at times of low power draw because the detection threshold is ~3 W per pole. We confirmed this accuracy of the RTM by comparing cumulative kWh readings from the RTM against those by the utility-provided electric meters used to bill residents. The comparison was carried out for a random sample of 78 apartments (20% of all apartments) and across a time period of 2–16 months between September 2017 and February 2019 (varied by building). We then determined the discrepancy in the real power measurements of the two metering systems as the relative difference of the RTM vs. the utility-meter measurement (using the kWh reading at the beginning and the end of the 2–16 months period). As shown in Fig. 3, the majority of discrepancies (49 of 78 apartments) are below ±1%, with only 4 of the discrepancies larger than ±5%, and 1 larger than ±10%. The average of the absolute value of all 78 discrepancies is 1.42%. As seen in Fig. 3, discrepancies higher than about 2% are limited to apartments with small average real powers. For example, the specific apartment with −11% discrepancy draws comparatively low average real power during the observation period (92 W average on the utility meter vs. 81 W average on the RTM). The small average indicates that a material portion of the total accumulated kWh in this apartment was likely incurred at real powers below the ~3 W detection threshold of the RTM, thus leading to the discrepancy. Communication with the superintendent of the respective building confirmed that the apartment in question was indeed unoccupied for a portion of the observation period. The power-weighted average of the absolute value of all 78 discrepancies is 0.92%, and thus consistent with the manufacturer’s rating of ±1% for the RTM^34^. As shown in Fig. 3, the RTM readings are above those of the utility-meter for some apartments but below for others. The average discrepancy, when observing the sign, is −0.17% (−0.05% when load-weighted). This shows that there is no material systematic error of the RTM when compared to the utility-meters.

### Data gaps

MFRED has a high degree of data completeness. MFRED comprises ~3.2 million 10-second time steps. For the vast majority of these (>99.9%), data were recorded for all 390 apartments and reported in MFRED for all 26 apartment groups (AG). In rare cases, meters for some (but never all) of the 390 apartments were offline, typically for one or a few 10-second time steps in a row. There is only one such case that lasted longer, namely on 09-July-2019 from 14:30 to 21:30 UTC. For time steps where any of the 390 meters were offline, the averaging of data into AGs was handled as follows: MFRED shows “NULL” for any time step and AG where fewer than the respective 15 meters were online (but the data of other AGs at the same time stamp is shown). This approach was chosen for three reasons: (i) to adhere to the 15/15 rule (see *Methods/Data preprocessing*); (ii) to avoid reporting “partial” data that would not have reflected all 15 apartments in a specific AG and whose values therefore would not have been comparable to those at other times for the same AG; and (iii) to leave possible interpolations of missing data to the discretion of MFRED users.

Completeness metrics for each individual csv file of MFRED are listed in Table 3, expressed as the fraction of the 390 meters that were online, averaged across all time steps in the specific file. Note that for the 15-min data, the only period with incomplete data (i.e., some of the 26 AGs show “NULL”) is on 09 July 2019; for all other 15-min time steps the data are complete.

Real power for those time steps and AGs showing “NULL” in MFRED can be interpolated as follows: Whenever meters were offline – and hence did not transmit their data to the *SQL* database – they nonetheless measured the cumulative electricity use (kWh). As a result, the kWh data record in MFRED immediately after a period of “NULL” for an AG correctly reflects the cumulative electricity that was consumed by the 15 apartments in that AG during the offline period. Therefore, the real power for any period shown as “NULL” in MFRED can be estimated via interpolation from the kWh data record immediately before and after the “NULL” period (or via more complex approaches preferred by MFRED users).

## Usage Notes

MFRED (5 csv files; 2.1GB) is publicly available via *Harvard Dataverse*^37^. Column headers and data records are explained in Tables 2 and 3. An overview of select possible uses and research applications of MFRED is listed in section *Background and summary*. Analyses presented here focus only on the characterization, validation, and benchmarking of the underlying data, in order to provide MFRED users with (i) a readily available overview and descriptive statistics of MFRED (Table 1, Figs. 1, 2); (ii) illustrations of seasonal variations and the use of real vs. reactive power for different end use types (Fig. 2); and (iii) the accuracy of the metering system (Fig. 3). Note that the analysis in Fig. 1 cannot be replicated with MFRED because the electricity use, floor area, and number of rooms of *individual* apartments are withheld in MFRED, in accordance with common data aggregation guidelines for utility data, specifically the 15/15 rule^36^ (see section *Data records/Apartment groups*). This aggregation has to be considered when using the dataset for said research applications, in particular for two of them: First, for disaggregation, the aggregate signal comprises on average more appliances (e.g., a refrigerator) and other individual end-uses (e.g., a specific light fixture in a specific room) at any given time than would be common for a typical, single apartment, thus requiring a successful disaggregation scheme to dissect the aggregate signal into more underlying components. To use a simple example, the aggregate signal of each apartment group comprises 15 separate refrigerators, each with its own on/off pattern. Second, for studying reactive power, the reactive power reported in MFRED is the average of the multiple appliances and other end-uses, not only in a single apartment but instead of those in 15 apartments. Because the reactive power of each appliance/end-use can range from negative to positive values, the reported aggregate reactive power can mask a wide range of the reactive powers of the increased number of individual underlying components.
